# Comparison of Cognitive Rehabilitation versus Donepezil Therapy on Memory Performance, Attention, Quality of Life, and Depression among Multiple Sclerosis Patients

**DOI:** 10.1155/2020/8874424

**Published:** 2020-11-22

**Authors:** Mohammad Mahdi Shahpouri, Majid Barekatain, Mahgol Tavakoli, Omid Mirmosayyeb, Ali Safaei, Vahid Shaygannejad

**Affiliations:** ^1^Isfahan Neurosciences Research Center, Alzahra Research Institute, Isfahan University of Medical Sciences, Isfahan, Iran; ^2^Department of Psychiatry, School of Medicine, Isfahan University of Medical Sciences, Isfahan, Iran; ^3^Department of Psychology, School of Educational Sciences and Psychology, University of Isfahan, Isfahan, Iran

## Abstract

**Background:**

Multiple sclerosis (MS) is a demyelinating disease of the central nervous system that affects cognitive performance and leads to depression and decreased quality of life (QOL). The current study aims to assess the effects of cognitive rehabilitation versus donepezil therapy on memory, attention, depression, and QOL in MS patients compared to placebo and control groups.

**Methods:**

Eighty MS patients were randomly selected from parallel randomized trials and divided into four groups: A: cognitive rehabilitation (10 sessions of 120 minutes), B: control (no intervention), C: donepezil (10 mg daily), and D: placebo. Patients received the intervention for three months. They were assessed for cognitive status, depression, and QOL prior to the intervention and immediately after that using abbreviated mental test (AMT), prospective and retrospective memory questionnaire (PRMQ), everyday memory questionnaire (EMQ), digit span, MSQOL-54, and second edition Beck depression inventory (BDI). We compared scores between groups after the intervention, as well as the progression of scores in every single group.

**Result:**

s. The cognitive rehabilitation group showed improvement in EMQ, RPMQ, digit span, physical and mental health subscales of MSQOL54, and depression (*P* < 0.05). We observed the same effect for donepezil except for the digit span test (*P* = 0.15). Intergroup comparison of scores showed the superiority of cognitive rehabilitation over donepezil in digit span, depression, and mental health scores.

**Conclusion:**

Both donepezil and cognitive rehabilitation effectively improve memory performance, attention, depression, and QOL in MS patients. Cognitive rehabilitation is superior altogether. This study is registered with the Iranian registry of clinical trials http://clinicaltrials.gov/ct2/show/IRCT2016042227522N1.

## 1. Introduction

Multiple sclerosis (MS) is a chronic demyelinating inflammatory disease that involves the central nervous system (CNS) and leads to the formation of focal lesions [1]. MS predominantly occurs in young females and causes a variety of physical, mental, and psychological disturbances that affect patients' life considerably [2, 3]. Memory, attention, performance, and processing speed are among the affected cognitive areas [4]. These disturbances added to the chronic nature of MS and patients' impaired motor function pose depression in affected individuals [5, 6]. Altogether, the patients' social, individual, and occupational lives will be affected and their quality of life (QOL) will get deteriorated [7].

Pharmacological interventions to improve cognitive impairment in MS patients are hardly investigated with conflicting findings. Donepezil, an acetylcholine esterase inhibitor (AChEI), is a neuroprotective drug used widely in the treatment of Alzheimer's Disease [8]. Moreover, it is an agonist of sigma receptors that can be used in the treatment of cognitive impairment [9]. Donepezil therapy has been associated with improved learning and memory function among MS patients in a previous study [10]. However, no superiority of donepezil over placebo has been observed in a larger multicenter study with regard to verbal memory [11].

On the other hand, cognitive rehabilitation has been suggested in affected MS patients more recently. In this technique, patients are trained to utilize more effective defensive mechanisms and give up impaired mechanisms to deal with their disabilities and worsened function [12]. The effect of cognitive rehabilitation on MS patients has been investigated in several studies with different designs and methodologies. While most previous investigators have focused on one or two cognitive domains, a few have utilized a more general approach [7]. Other differences include variations in intervention duration and frequency, utilizing a similar approach for all patients or tailoring them with respect to the individual's impairments, and evaluating the effect on different cognitive aspects [7]. While some studies suggest that cognitive rehabilitation could improve quality of life, memory, fatigue, depression, and attention, others report negative findings [4, 7, 13–15]. Therefore, there is still a need to investigate it more.

In addition to cognitive rehabilitation, pharmacological interventions are also understudied here. It is yet to be cleared whether these approaches are superior to one another. Here, we aimed to assess the effect of cognitive rehabilitation and donepezil therapy on memory, attention, quality of life, and depression among patients with multiple sclerosis compared to two control groups, one receiving placebo and the other one not receiving any intervention. Our main goal was to find out which intervention is superior to other options.

## 2. Methods

### 2.1. Study Population

The current study is a prospective study on subjects from two parallel double-blind randomized clinical trials (one published [16] and one in copyediting) conducted on 80 patients referred to Multiple Sclerosis Clinic of Kashani Hospital, affiliated to Isfahan University of Medical Sciences, in 2018. The eligibility criteria were patients ≥18 years with the diagnosis of MS based on the McDonald criteria [17], with Expanded Disability Status Scale (EDSS) [18] ≤5.5, mild/moderate memory impairment (assessed in the screening phase using the questionnaires explained below), who were able to read and write. Eligible subjects were invited to participate in the screening phase by completing everyday memory questionnaire 28-item scale [19] and Beck depression inventory [20] to assess their cognitive ability and depression state. Patients were excluded if they had severe memory impairment (assessed by questionnaires in the screening phase explained below), severe depression, other neurological disorders, history of using AChEIs or psychostimulants, abnormal hepatic or kidney function, current use of anticholinergic medications, or known psychiatric illnesses. We should note that patients needed visual and auditory abilities to complete some tests, so those with severe visual or auditory deficiencies were not included in the study.

### 2.2. Sample Size and Recruitment

At the power of 80% and a significance level of 0.05, 20 patients were calculated to be included in each group. Written informed consent was obtained from all participants prior to the screening phase. The study was approved by the ethics committee of Isfahan University of Medical Sciences (registration code: 398611) and was registered in the Iranian registry of clinical trials http://clinicaltrials.gov/ct2/show/IRCT2016042227522N1.

### 2.3. Study Protocol

This study was conducted along with two larger studies (donepezil versus placebo and cognitive rehabilitation versus control). Given the calculated sample size, groups of 20 subjects were randomly selected from larger groups in parallel studies. For the larger studies, participants have been randomly assigned to study groups using random allocation software. On those parallel studies, a unique number was assigned to each subject and they were grouped based on those numbers. Numbers were previously divided between study groups. Both patients and interviewers were blinded to the randomization status. The study groups in the current study were group A (cognitive rehabilitation), group B (control without intervention, called control in this manuscript), group C (donepezil treatment), and group *D* (placebo, called placebo in this manuscript).

Patients were asked to complete the following questionnaires prior to intervention: Abbreviated Mental Test (AMT) [21], Prospective and Retrospective Memory Questionnaire (PRMQ) [22], Multiple sclerosis Quality of Life 54 (MQOL54) [23], and digit span test for attention assessment [24]. Also, they had completed the everyday memory questionnaire 28-item scale [19] and Beck depression inventory [20] in the screening phase as mentioned previously.

In group A, the therapeutic intervention was 10 sessions of cognitive rehabilitation courses. Each session lasted for 2 hours and was individualized for each case based on impaired function reconstruction and modulation. Sessions were held every 7–10 days. Generally, in each class, the therapist aimed for reinforcement and/or consolidation of previous cognitive abilities which have been impaired and tried to reinforce other remained abilities for compensation of impaired abilities. In this way, patients could rehabilitate their role in society and actively maintain their functions [12].

Patients in group C received 10 mg of donepezil (Yasnal®; KRKA pharmaceutical company, Slovenia) daily for three months. Patients in group *D* received placebo pills daily for three months. Placebo pills were similar to donepezil tablets in shape, color, and packaging (Pharmacy Faculty; Isfahan University of Medical Sciences). Patients in group B did not receive any intervention but were followed for three months like other groups.

At the end of the intervention, patients were asked to complete all the questionnaires again. Of note, all patients continued their disease-modifying MS treatment during the study.

### 2.4. Questionnaires

The Persian version of AMT includes 10 questions that evaluate cognitive impairment. The questions ask for age, orientation for time, date, and place, occupation, date of birth, questions about the country, invert counting, and repeating an address. The reliability of this test determined to be 0.89 PRMQ consisted of 16 items that target different types of memory failure, as well as the resultant frustration. The items are scored on a 5-point scale (1–5) with higher scores accounting for more frequent memory failures. The reliability of the Persian version is reported to be 0.80 for the Persian version [22]. Everyday memory questionnaire consists of 28 items, and each item scores from 1 to 9. The higher the score, the more the memory dysfunction. This questionnaire assesses patients' memory during the recent six months. Its Persian version was validated by Sharifi et al. in 2007 [25].

MSQOL consisted of 54 questions assessing physical health, role limitation due to physical and emotional problems, pain, emotional well-being, energy, health perception, social function, cognitive function, health distress, sexual function, and overall quality of life. The higher total scores demonstrate better quality of life in the target subset of the questionnaire. The reliability of the Persian version is reported to be 0.96 [23].

Digit span test evaluates working memory and attention by asking the patients to repeat a list of digits that is being read for them. Higher scores show a greater number of numbers and, therefore, better working memory function [26].

The second version of BDI measures the severity of depressive symptoms. It includes 21 items scoring from 0 (absent or mild) to 3 (severe) and the greater scores show more severe depression. The reliability of the Persian version of the test is 0.84 [20].

### 2.5. Cognitive Rehabilitation Protocol

The cognitive rehabilitation agenda included topics on attention, concentration, visual and auditory memory, and autobiography memory. We modified the approaches according to the severity of cognitive impairment, with the aim of optimizing the residual functions. We utilized the mnemonic approach that includes visual imagery, theological organization and relational strategies (including mnemonics of fiction), the clues about the first word, chain connection, and the technique of PQRST (Preview, Question, Read, Self-recitation, and Test) [27–29]. A total of 10 sessions were held for participants in groups, each session for 120 minutes. In these sessions, the instructor followed the agenda by explaining and discussing the memory and related disturbances in daily life, followed by discussions on the autobiographical memory, its subtypes, and disturbances. Participants were also trained for the technique of recalling positive memories through autobiographical memory, followed by practicing on samples and sharing the positive recalls. We should note that the cognitive rehabilitation method was designed based on the previous literature as well as the expertise and skills of our therapists and psychology team. We specifically tried to utilize the approaches that our personnel were better at delivering them instead of using other suggestions but delivering them imperfectly.

### 2.6. Outcome Measures

The primary outcome was cognitive function, measured by EMQ, AMT, PRMQ, and “digit span test”. We powered the study for digit span test results, but it could also be applied to other questionnaires on cognitive function. The secondary outcomes were quality of life and depression, measured by MSQOL and BDI, respectively.

### 2.7. Study Analysis

Descriptive data were presented in mean and frequency (%). After assessing data in each category/group for normality of distribution, appropriate statistical tests were selected. We used a paired *t*-test or Wilcoxon signed-rank test to compare interval variables in the same groups before and after the intervention. Multivariate analysis of covariance (MANCOVA) was used to compare scores after intervention between study groups with adjustment for the baseline data. We also used a post hoc test of Bonferroni in case of any observed differences.

We should note that the samples from two parallel studies were statistically comparable to each other, because the studies were designed with a similar methodology, had similar timing, and looked for similar outcome measures. We carried out statistical analysis using SPSS version 24 (Armonk, NY). A *P* value of less than 0.05 was considered significant.

## 3. Results

As stated before, we selected twenty subjects in each group from the pool of patients gathered in this study and two parallel substudies. Baseline data of patients in four study groups are summarized in Table 1. We found no statistically significant difference between groups with regard to demographic features and disease characteristics (duration, disease-modifying agents, dominant symptoms, and annualized relapse rate) (*p* > 0.05).

**Figure 1:**
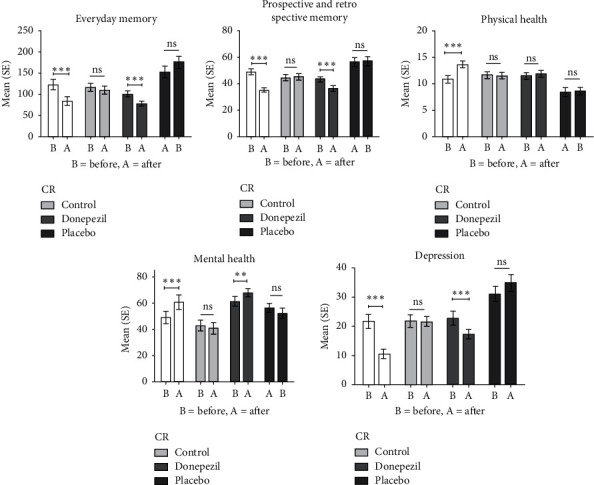
Comparison of pretests-posttests of cognitive rehabilitation, control, donepezil and placebo on memory performance, attention, quality of life, and depression in multiple sclerosis patients. CR: cognitive rehabilitation; ns: not significant. *∗P* < 0.05, *∗∗P* < 0.01, *∗∗∗P* < 0.0001.

Figure 1 demonstrates the mean and standard error (SE) of study questionnaires before and after intervention in four study groups. We found statistically significant improvement with regard to PRMQ, EMQ, QOL (both mental and physical health subscales), and depression in cognitive rehabilitation and donepezil groups (*p* < 0.001). However, digit span scores improved in the cognitive rehabilitation group only (*p* < 0.05). Results from MANCOVA are presented in Table 2.

Table 3 shows the post hoc analysis of study tools between groups two-by-two. We found no statistically significant difference comparing pretest and posttest scores between placebo and control groups per post hoc analysis (*p* > 0.05). Cognitive rehabilitation was superior to donepezil therapy with respect to improving digit span test scores, mental health subscale of quality of life, and depression (*p* < 0.05). However, both cognitive rehabilitation and donepezil improved EMQ, PRMQ, and physical health subscale of quality of life similarly (*p* > 0.05). Compared to the placebo and control groups, cognitive rehabilitation improved the outcome in all questionnaires (*p* < 0.05). But donepezil therapy failed to improve digit span scores compared to either placebo or control group, as well as EMQ scores compared to the control group.

With respect to adverse events, two patients in the placebo group reported mild nausea following taking pills. In the donepezil group, two patients reported transient headache, one nausea, and one diarrhea. All symptoms were self-limited.

## 4. Discussion

Multiple sclerosis affected patients are prone to cognitive impairment; they may also experience conditions such as depression that would additionally affect their quality of life [4, 6]. Decelerating the disease progression and facilitating recovery through medical interventions are necessary to help them overcome the psychological burden of the disease and become motivated to adapt to their new condition and regain their functionality [30]. The latter, however, is not completely achieved through the management of neurological symptoms and may require more interventions focusing on psychological and cognitive impairments.

In the current study, we tried to assess the effectiveness of previously proposed techniques for cognitive impairment compared to each other and control groups. Although memory performance is the most common cognitive function impaired in MS patients [31], we tried to cover other aspects of cognitive and memory function as well by utilizing different questionnaires in the assessment phase. Based on our findings, both cognitive rehabilitation and donepezil therapy successfully improved memory performance, attention, quality of life, and depression, although we found no difference in digit span scores between donepezil and control/placebo groups. Taken together, we can conclude that cognitive rehabilitation is superior to donepezil therapy in regaining function in the mentioned areas.

To explain why donepezil could possibly improve memory function, we should address the major role of the hippocampus in human memory and its neuronal networks rich in cholinergic neurons. With the same mechanism, injuries in muscarinic cholinergic endpoints are responsible for memory disruption in dementia patients [32, 33]. Consequently, donepezil as an AChEI could improve memory function in MS patients as it does in Alzheimer's Disease. However, it has not always been the case in previous reports. For example, Hughes et al. reported no encouraging outcomes following donepezil therapy in a group of MS patients while they found cognitive rehabilitation to be an effective intervention here [34]. Similarly, Krupp et al. reported no positive effect of donepezil on memory performance [11]. In contrast, some other researchers have claimed a promising outcome of donepezil on memory in MS patients [35, 36]. For example, in another double-blinded randomized clinical trial, Krupp et al. showed that a twenty-four-week treatment with donepezil was superior to placebo in the rehabilitation of memory performances [36].

The favorable outcome of cognitive rehabilitation has also been reported on autobiographical memory [37]. Moreover, they found changes in posterior fossa following cognitive rehabilitation, which was also confirmed by Raskin et al. in another study [38]. Also, it is shown that cognitive rehabilitation could improve attention and executive function [39]. Per our findings, we believe that both interventions are effective in improving cognitive impairment although cognitive rehabilitation is the superior one. Similar findings have also been reported in other studies [40, 41].

On the other hand, depression is a major complaint of MS patients which is also associated with cognitive impairment; also, it is not well established. For instance, a study reported that frontal lobe functioning is compromised in depressed cases while this lobe is responsible for cognitive performances such as attention, concentration, and information processing [42]. Bruce et al. suggested the association of depression with memory impairment, as well. Comparing MS patients with and without depression, they found that depressed cases estimate their everyday memory in proportion to the actual performance while those without depression overestimate their ability [43]. We also hypothesized that improving cognitive function through cognitive rehabilitation could also improve depression in affected individuals and observed the desired outcome.

Quality of life is another area significantly affected in MS patients. Decreased quality of life occurs due to physical disabilities, long-term duration of disease, fatigue, and depression, as well as other impairments, including memory and cognition [44, 45]. A study by Birnboim et al. showed that cognitive rehabilitation improved MS patients' quality of life. The authors suggested that the higher self-esteem achieved following rehabilitation is a possible factor in improving quality of life [46].

Comparing the placebo and the control group (not receiving any intervention), we find improved everyday memory and depression in the placebo group. The effectiveness of placebo in psychological problems has been known in various disorders. Although donepezil improved both of these categories compared to placebo, it may still suggest that some observed findings here have been due to the placebo effect.

Our study was limited by the relatively small number of subjects in each group, lack of long-term follow-ups to evaluate the persistency of observed outcomes, and not having groups receiving combined interventions or using cross-over design to assess the effectiveness of interventions more precisely. We randomly selected participants from a larger pool of patients recruited in parallel randomized clinical trials, which would probably not benefit from all strengths of randomized trials. Also, our study lacks an active control group for cognitive rehabilitation. Another limitation is relying on a self-report memory scale to include patients in the study. Moreover, almost all the cognitive measures investigated in this study could be potentially confounded by depression and other psychosocial factors, although we have tried to control for the outcome measures for baseline scores using the appropriate statistical methods. Despite limitations, it presents useful and practical information with respect to cognitive impairment in MS cases, investigating various aspects of cognition at the same time.

## 5. Conclusion

To conclude, both cognitive rehabilitation and donepezil therapy could effectively improve memory performance, attention, depression, and quality of life in MS patients. However, cognitive rehabilitation is superior to donepezil, especially when it comes to depression and digit span memory. We recommend studies on combined methods of intervention in the future.

## Figures and Tables

**Table 1:** Baseline demographic and clinical characteristics.

	CR *n* = 20	Control *n* = 20	Donepezil *n* = 20	Placebo *n* = 20	*P* value
Demographic characteristics					
Age, mean ± SD (years)	32.2 ± 8.4	30.6 ± 7.0	31.6 ± 6.8	30.7 ± 7.4	0.89
Education, *n* (%)					
High school	2 (10)	3 (15)	3 (15)	1 (5)	0.810
Bachelors	15 (75)	11 (55)	13 (65)	14 (70)	
Postgraduates	3 (15)	6 (30)	4 (20)	5 (25)	
Disease characteristics					
EDSS, mean ± SD	2.67 ± 1.31	2.55 ± 1.39	2.75 ± 1.14	2.62 ± 1.32	0.969
MS subtypes, *n* (%)					
RRMS	16 (80)	17 (85)	17 (85)	18 (90)	0.911
SPMS	2 (10)	3 (15)	2 (10)	2 (10)	
PPMS	2 (10)	0 (0)	1 (5)	0 (0)	
Disease duration, mean ± SD (years)	7.20 ± 4.21	6.65 ± 3.56	6.85 ± 3.56	5.90 ± 3.27	0.697
Disease-modifying agents, *n* (%)					0.875
Interferon beta	15 (75)	17 (85)	16 (80)	17 (85)	
Fingolimod	3 (15)	2 (10)	3 (15)	3 (15)	
Rituximab	2 (10)	1 (5)	1 (5)	0 (0)	
Annualized relapse rate, *n* (%)					0.983
0	14 (70)	16 (80)	15 (75)	16 (80)	
1	4 (20)	3 (15)	4 (20)	3 (15)	
2	2 (10)	1 (5)	1 (5)	1 (5)	
Dominant symptoms					
Motor symptoms	9 (45)	7 (35)	8 (40)	8 (40)	0.989
Balance symptoms	4 (20)	4 (20)	4 (20)	3 (15)	
Sensory symptoms	3 (15)	4 (20)	4 (20)	4 (20)	
Sphincter symptoms	2 (10)	2 (10)	2 (10)	1 (5)	
Visual symptoms	2 (10)	3 (15)	2 (10)	4 (20)	

SD: standard deviation; CR: cognitive rehabilitation, MS: multiple sclerosis, EDSS: Expanded Disability Status Scale; RR: relapsing remitting; SP: secondary progressive; PP: primary progressive.

**Table 2:** Results from multivariate analyses of covariance (MANCOVA) comparing scores of posttests between groups.

	CR estimate (CI 95%)	Control estimate (CI 95%)	Donepezil estimate (CI 95%)	Placebo estimate (CI 95%)	*P* value
Everyday memory	83.89^a^ (66.9, 100.7)	112.84 (92.6, 133.0)	93.78 (74.1, 113.4)	156.84 (135.5, 178.1)	*F* (3, 70) = 9.93 *d* = .336, *P* < 0.001
Prospective and retrospective memory	35.41 (31.9, 38.8)	48.76 (44.6, 52.8)	38.22 (34.2, 42.2)	51.79 (47.4, 56.1)	*F* (3, 70) = 16.08 *d* = 0.45, *P* < 0.001
Digit span memory	13.43 (12.6, 14.2)	10.77 (9.8, 11.7)	11.18 (10.2, 12.1)	10.35 (9.3, 11.3)	*F* (3, 70) = 10.41 *d* = 0.34, *P* < 0.001
Physical health	65.24 (60.4, 70.0)	49.99 (44.2, 55.7)	60.32 (54.7, 65.8)	49.56 (43.5, 55.5)	*F* (3, 70) = 8.44 *d* = 0.30, *P* < 0.001
Mental health	68.26 (62.1, 74.4)	45.43 (38.0, 52.8)	56.04 (48.8, 63.2)	36.78 (29.0, 44.5)	*F* (3, 70) = 15.58 *d* = 0.44, *P* < 0.001
Depression	11.74 (8.7, 14.6)	24.27 (20.7, 27.8)	18.46 (15.0, 21.9)	29.86 (26.1, 33.5)	*F* (3, 70) = 21.82 *d* = 0.52, *P* < 0.001

^a^ Prediction adjusted with baseline covariate model MANCOVA; CR: cognitive rehabilitation; CI 95%: confidence interval 95%; *d*: hesitantly defined effect sizes as “small, *d* = 0 .2,” “medium, *d* = 0 .5,” and “large, *d* = 0.8,”

**Table 3:** Individual post hoc analyses of MANCOVA to find group differences among cognitive rehabilitation, donepezil, placebo, and control.

	CR versus Control		CR versus Donepezil		CR versus Placebo		Donepezil versus Control		Donepezil versus placebo		Placebo versus Control	
	MD (SE)	*P* value^b^	MD (SE)	*P* value	MD (SE)	*P* value	MD (SE)	*P* value	MD (SE)	*P* value	MD (SE)	*P* value
Everyday memory	−28.95^a^ (13.12)	0.031	−9.89 (12.95)	0.448	−72.95 (13.81)	<0.001	−19.06 (14.50)	0.194	−63.06 (15.37)	<0.001	43.99 (15.37)	0.006
Prospective and retrospective memory	−13.34 (2.66)	<0.001	−2.80 (2.63)	0.291	−16.37 (2.83)	<0.001	−10.54 (2.95)	0.001	−13.56 (3.08)	<0.001	3.02 (3.13)	0.337
Digit span memory	2.66 (0.62)	<0.001	2.25 (0.61)	0.001	3.08 (0.66)	<0.001	0.41 (0.69)	0.553	0.83 (0.72)	0.254	−0.42 (0.73)	0.57
Physical health	15.25 (3.70)	<0.001	4.92 (3.66)	0.185	15.68 (3.92)	<0.001	10.33 (4.10)	0.015	10.76 (4.28)	0.015	−0.43 (3.66)	0.921
Mental health	22.833	<0.001	12.223	0.012	31.483	<0.001	10.610	0.049	19.260	0.001	−8.65	0.128
Depression	−12.530 (2.68)	<0.001	−6.726 (2.63)	0.004	−18.12 (2.32)	<0.001	−5.80 (3.21)	0.026	−11.40 (2.54)	<0.001	5.59 (2.69)	0.04

MANCOVA: multivariate analysis of covariance; MD: mean difference; SE: standard error; CR: cognitive rehabilitation, based on estimated marginal means MANCOVA. ^b^ Adjustment for multiple comparisons: Bonferroni.

## Data Availability

Data are available upon request to the corresponding author.
